# The Thr92Ala polymorphism in the type 2 deiodinase gene is linked to depression in patients with COVID-19 after hospital discharge

**DOI:** 10.3389/fendo.2024.1366500

**Published:** 2024-06-07

**Authors:** Daniele Carvalhal de Almeida Beltrão, Fabyan Esberard de Lima Beltrão, Giulia Carvalhal, Fabyanna Lethicia de Lima Beltrão, Amanda da Silva Brito, Hatilla dos Santos Silva, Helena Mariana Pitangueira Teixeira, Juliana Lopes Rodrigues, Camila Alexandrina Viana de Figueiredo, Ryan dos Santos Costa, Liana Clebia De Morais Pordeus, Giciane Carvalho Vieira, Helton Estrela Ramos

**Affiliations:** ^1^ Postgraduate Program in Cognitive Neuroscience and Behavior, Center for Health Sciences, Federal University of Paraíba, João Pessoa, Paraíba, Brazil; ^2^ Department of Internal Medicine, University Centre of João Pessoa (UNIPE), João Pessoa, Paraíba, Brazil; ^3^ Department of Internal Medicine, Lauro Wanderley University Hospital, Federal University of Paraíba, João Pessoa, Paraíba, Brazil; ^4^ Center for Biological and Health Sciences, Federal University of Campina Grande, Campina Grande, Paraíba, Brazil; ^5^ Post-Graduate Program in Medicine and Health, Medical School of Medicine, Federal University of Bahia, Salvador, Brazil; ^6^ Department of Internal Medicine, Royal Portuguese Hospital of Beneficence, Recife, Pernambuco, Brazil; ^7^ Bioregulation Department, Health and Science Institut, Federal University of Bahia, Salvador, Bahia, Brazil; ^8^ Laboratory of Immunopharmacology and Molecular Biology, Health Sciences Institute, Federal University of Bahia, Salvador, Bahia, Brazil; ^9^ Postgraduate Program in Interactive Processes of Organs and Systems, Health and Science Institute, Federal University of Bahia, Salvador, Bahia, Brazil

**Keywords:** post-COVID-19, Thr92Ala-DIO2 polymorphism, thyroid function, biomarkers, depression

## Abstract

**Background:**

The Thr92Ala-DIO2 polymorphism has been associated with clinical outcomes in hospitalized patients with COVID-19 and neuropsychiatric diseases. This study examines the impact of the Thr92Ala-DIO2 polymorphism on neuropsychological symptoms, particularly depressive symptoms, in patients who have had moderate to severe SARS-CoV-2 infection and were later discharged.

**Methods:**

Our prospective cohort study, conducted from June to August 2020, collected data from 273 patients hospitalized with COVID-19. This included thyroid function tests, inflammatory markers, hematologic indices, and genotyping of the Thr92Ala-DIO2 polymorphism. Post-discharge, we followed up with 68 patients over 30 to 45 days, dividing them into depressive (29 patients) and non-depressive (39 patients) groups based on their Beck Depression Inventory scores.

**Results:**

We categorized 68 patients into three groups based on their genotypes: Thr/Thr (22 patients), Thr/Ala (41 patients), and Ala/Ala (5 patients). Depressive symptoms were less frequent in the Thr/Ala group (29.3%) compared to the Thr/Thr (59.1%) and Ala/Ala (60%) groups (*p* = 0.048). The Thr/Ala heterozygous genotype correlated with a lower risk of post-COVID-19 depression, as shown by univariate and multivariate logistic regression analyses. These analyses, adjusted for various factors, indicated a 70% to 81% reduction in risk.

**Conclusion:**

Our findings appear to be the first to show that heterozygosity for Thr92Ala-DIO2 in patients with COVID-19 may protect against post-COVID-19 depression symptoms up to 2 months after the illness.

## 1 Introduction

Since the onset of the COVID-19 pandemic on 11 March 2020, concerns about the increased risk of neuropsychiatric disorders among survivors have risen. Long-term COVID, involving post-acute sequelae after SARS-CoV-2 infection, can lead to various dysfunctions of extrapulmonary organs, including neuroinflammation, which may contribute to the development of depression (1, 2).

The peripheral cytokines may potentially affect brain function through direct action or via afferent pathways. Individuals with autoimmune diseases and severe infections are more likely to experience depression, and therapeutic cytokines can trigger depressive symptoms. The cytokines under extensive study include interleukin (IL) (IL-1β, IL-5, IL-6, IL-12, and IL-17), tumor necrosis factor (TNF), and interferons (IFNs), representing the inflammatory aspect, and IL-10, associated with resolution (3).

Depression is a complex polygenic disorder influenced by environment. Depending on various studies, its heritability ranges from 30% to 50%, with stress and imbalances in the HPA axis being notable contributing factors. Genetic studies often neglect stress effects, contributing to inconsistent results (4, 5).

There is a statistically significant association between thyroid dysfunction and the development of mental distress, mood disorders, and depression (5, 6). A comprehensive meta-analysis of 12,315 individuals indicated that patients with subclinical hypothyroidism have a higher risk of depression compared to euthyroid controls (relative risk of 2.35, 95% CI: 1.84 to 3.02, *p* < 0.001) (7). Depression-related thyroid hormone (TH) level changes include increased reversed triiodothyronine (rT3) (8, 9) and decreased circulating T3 and TSH levels (10, 11).

The DIO2 gene encodes type 2 deiodinase (D2), a crucial enzyme in converting the pro-hormone T4 into its active form, T3. The Thr92Ala-DIO2 polymorphism is found in approximately half of the global population and has been linked to chronic diseases such as type 2 diabetes mellitus (12, 13), obesity (14), arterial hypertension (15), osteoporosis (16), mental distress (17, 18), and depression (19, 20).

Lately, we investigated 220 consecutive patients with moderate to severe COVID-19 that showed a protective role of the heterozygous state of the polymorphic variant DIO2 (Thr92Ala) in mortality and severity from COVID-19. The heterozygous genotype (Thr/Ala) was associated with a 47%–62% reduced in-hospital risk. The protective role of Thr92Ala’s heterozygous advantage was supported in a meta-analysis of 21 studies in more than 20,000 patients with diseases such as diabetes, obesity, ischemic stroke, myocardial infarction, and left ventricular hypertrophy (21).

This study aims to investigate the potential correlation between the Thr92Ala-DIO2 polymorphism and depressive symptoms 2 months after COVID-19 hospital discharge. Additionally, it aims to explore various metabolic and hormonal biomarkers alongside tomographic measurements evaluated upon hospital admission. The results of this study could help stratify patients and enable early identification of neuropsychiatric disorders in COVID-19 survivors, facilitating future interventions.

## 2 Materials and methods

**Figure 1:**
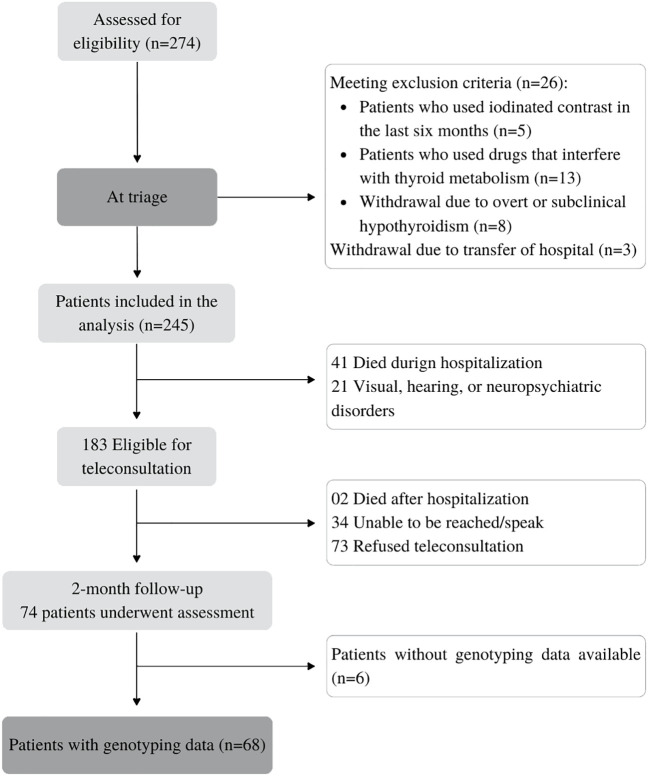
Flowchart of the study.

This research was a branch of a broader prospective, longitudinal cohort study, designed to assess thyroid dysfunction in patients with moderate to severe COVID-19 requiring intensive or semi-intensive care. We evaluated 273 consecutive patients hospitalized with COVID-19 between June and August 2020 at the Hospital Metropolitano Dom José Maria Pires (a tertiary referral hospital for COVID-19) in João Pessoa, Paraíba, Brazil. Following discharge, 78 patients were assessed as outpatients for neuropsychiatric issues (**Figure 1**). Ethical approval was granted by the Hospital Universitário Lauro Wanderley’s Ethics Committee for Human Research (CAAE:31562720.9.0000.5183).

Inclusion criteria involved patients who tested positive for SARS-CoV-2 through quantitative real-time reverse transcription polymerase chain reaction (rRT-qPCR—Biomol OneStep/COVID-19, IBMP, Paraná, Brazil) with respiratory tract samples. In cases of negative rRT-qPCR, eligibility was determined based on clinical, radiological, and serological parameters (positive IgG for SARS-CoV-2). Exclusion criteria comprised patients with a history of thyroid disease or the use of drugs affecting thyroid metabolism, pregnancy, personal neuropsychiatric disorders, and the use of neuropsychiatric medications.

The primary outcomes were long-term depression symptomatology in previously hospitalized COVID-19 survivors according to the Thr92Ala-DIO2 polymorphism. Secondary outcomes were blood biochemistry, thyroid function tests, length of stay, comorbidities, complications, and severity scores according to Thr92Ala-DIO2 polymorphism and depressive symptomatology.

The research physicians gathered detailed clinical information on each patient within 48 h of admission using a standard questionnaire and severity scales (qSOFA and NEWS2). After discharge, patients were contacted and invited (30–45 days after discharge) by telephone for a medical consultation at the HULW, to evaluate post-COVID-19 symptoms and signs, including depression symptoms through Beck Depression Inventory (BDI). The BDI is a self-assessment instrument composed of 21 items with scores ranging from 0 to 63. The cutoff scores for the BDI were ≤9 (without depression) and >10 (with depression) (22).

Blood samples (50 mL) were collected within the first 48 h of hospital admission (before any interventions or therapy, including steroids and heparin). Laboratory tests performed included interleukin 6, D-dimer, alanine aminotransferase (ALT), aspartate aminotransferase (AST), creatinine, high-sensitivity C-reactive protein (hs-PCR), and lactate dehydrogenase (LDH). The method used in all examinations was automated chemiluminescence (MAGLUMI-2000-PLUS; Shenzhen New Industries Biomedical Engineering Co., Shenzhen, China). The complete blood cell count with differential was performed on a MEK-7300 hematological analyzer (Nihon Kohden^®^, Tokyo, Japan).

Patients underwent chest CT to diagnose suspected SARS-CoV-2 pneumonia (ground-glass opacity, mosaic attenuation, and consolidation). A semiquantitative CT severity score proposed by Pan et al. was used in all cases (23).

Genomic DNA was extracted from peripheral blood leukocytes using standard techniques. In this study the polymorphism was determined by the TaqMan^®^ SNP Genotyping method (7500 Real-Time PCR Systems, Applied Biosystems, Foster City, CA, USA), using the assay for genotyping with TaqMan^®^ probes and primers, in a combination of hybridization and DNA polymerase activity, associated with fluorescence detection (24). We used the software Sequence Detection, version 1.3 (Applied Biosystems, CA) to analyze the data.

Statistical analysis: To determine the requisite sample size, we employed GPower 3.1.9.7 software, setting the significance level of α = 0.05, the desired statistical power of 0.95, and the effect size (F2) of 0.10. The outcome indicated a minimum sample size of 158 patients from the initial 273. Using the Cochran formula with finite population correction (population size *n* = 273) and aiming for a 95% confidence level and a margin of error within ±10% for prevalence estimation, the analysis required a minimum of 53 patients. Thus, our study group of 68 patients was sufficiently large for a comprehensive analysis.

The data were represented as the median ± interquartile range (IQR). Non-parametric tests were used for quantitative analysis, including the Mann–Whitney test for two-variable comparisons, and the Kruskal–Wallis test with Dunn’s *post hoc* test for multiple comparisons. Chi-square or Fisher’s exact test was used for qualitative analyses. Spearman’s rank correlation coefficient assessed the linear association between variables. Univariate and multivariate logistic regression analyses evaluated the risk of post-hospital discharge depressive symptoms among patients.

## 3 Results

Of the 274 adult patients admitted with COVID-19 to a referral hospital, 183 were initially selected for post-discharge assessment. Eligibility assessment led to the exclusion of 109 individuals: 2 due to post-hospitalization death, 34 were unreachable, and 73 declined participation. Furthermore, six patients were excluded due to incomplete genotype data, resulting in a final cohort of 68 patients (**Figure 1**).

The group of 68 patients was stratified into three subgroups based on genotype: Thr/Thr (*n* = 22), Thr/Ala (*n* = 41), and Ala/Ala (*n* = 5) (**Figure 1**). The Thr allele frequency was 0.62 and the Ala allele frequency was 0.37, with a distribution that was in Hardy–Weinberg equilibrium (*p* = 0.07; chi-square test). Baseline sociodemographic and clinical characteristics are summarized in **Table 1**. During follow-up, the median age of patients was 54.5 (45–67) years, 27 patients were over 60 years old (39.7%), and 48 patients (70.6%) were men. The median length of stay of patients in the hospital was 6 days (4.2–8), and seven patients (10.3%) were admitted to the ICU (**Table 1**).

**Table 1:** Demographic and clinical characteristics of the patient cohort and their association with Thr92Ala-DIO2 polymorphism and depressive symptomatology (*n* = 68).

Variables	Total	Thr/Ala	Thr/Thr + Ala/Ala	*p*-value	Non-depression	Depression	*p*-value
	(*n* = 68)	(*n* = 41)	(*n* = 27)		(*n* = 40)	(*n* = 28)	
** Age (years), median (IQR)**	54.5 (45–67)	53 (42–65)	59 (50–68)	0.192	52 (42–59)	65.5 (53–72)	0.004
** Age > 60 years, *n* (%)**	27 (39.7)	15 (36.6)	12 (44.4)	0.516	**10 (25)**	**17 (60.7)**	**0.0052**
** BMI (kg/m²)**	32 (27–36)	29.8 (26–35)	32.8 (30–38)	0.068	30.7 (28–35)	32.7 (26–38)	0.455
** Gender male, *n* (%)**	48 (70.6)	31 (75.6)	32.8 (30–38)	0.262	**34 (85)**	**14 (50)**	**0.0028**
** Day to symptom (days)**	9 (7–11)	10 (7–11)	9 (7–10)	0.295	9 (7–10.7)	10 (6.2–11)	0.805
Associated morbidities							
** Hypertension, *n* (%)**	43 (63.2)	25 (61)	18 (66.7)	0.633	22 (55)	21 (75)	0.126
** Diabetes, *n* (%)**	29 (42.6)	16 (39)	13 (48.1)	0.456	15 (37.5)	14 (50)	0.330
** Obesity, *n* (%)**	**40 (58.8)**	**19 (46.3)**	**21 (77.8)**	**0.01**	22 (55)	18 (64.3)	0.466
** Cardiopathy, *n* (%)**	7 (10.3)	3 (7.3)	4 (14.8)	0.319	2 (5)	5 (17.9)	0.115
** Chronic pneumopathy (%)**	3 (4.4)	1 (2.4)	2 (7.4)	0.329	0 (0)	3 (10.7)	0.065
Complications							
** Use of vasoactive drugs, *n* (%)**	2 (2.9)	1 (2.4)	1 (3.7)	0.762	0 (0)	2 (7.1)	0.165
** Length of hospital stay (days), (IQR)**	6 (4.2–8)	6 (4.5–8)	5 (4–10)	0.636	6 (4.2–8)	6 (4.2–9.5)	0.839
** ICU admission, *n* (%)**	7 (10.3)	4 (9.7)	3 (11.1)	0.857	2 (5)	5 (17.9)	0.115
Scores systems							
** BDI score, median (IQR)**	**8 (3.2–14)**	**7 (3–10)**	**10 (5–22)**	**0.032**	**4.5 (2.2–7)**	**16 (11.2–26)**	**<0.0001**
** NEWS2 score, median (IQR)**	5.5 (5–7)	6 (5–7.5)	5 (5–6)	0.544	6 (5–7)	5 (5–7)	0.478
** q-SOFA score, median (IQR)**	1 (0–1)	1 (0–1)	1 (1–1)	0.672	1 (0.25–1)	1 (0–1)	0.423
** CT COVID score, median (IQR)**	20 (15–20)	20 (15–20)	20 (15–20)	0.958	20 (15–20)	20 (15–20)	0.323

Mann–Whitney test was performed for continuous variables (age, NEWS2, qSOFA, and TC COVID Score) while Fisher’s exact test was performed for all other variables.

BDI, Beck’s Depression Inventory; BMI, body mass index; ICU, intensive care unit; IQR, interquartile range; NEWS2, National Early Warning Score 2; NTIS, Non-thyroidal Illness Syndrome; q-SOFA, quick sepsis related organ failure; CT COVID, Chest computed tomography score in COVID-19 patients. The bold values correspond to the results assessed with significant p-values (P < 0.01).

Risk factors for post-discharge depressive symptoms were analyzed using Mann–Whitney and Fisher’s tests. There was no significant difference between the risk factors (hypertension, diabetes mellitus, obesity, and heart disease), complications (use of vasoactive drugs, admission to the ICU, and hospital stay), and severity scores (NEWS2, q-SOFA, and CT-COVID). A higher percentage of non-depressed patients were younger than 60 (75%) and were men (85%) (**Table 1**).

**Figure 2:**
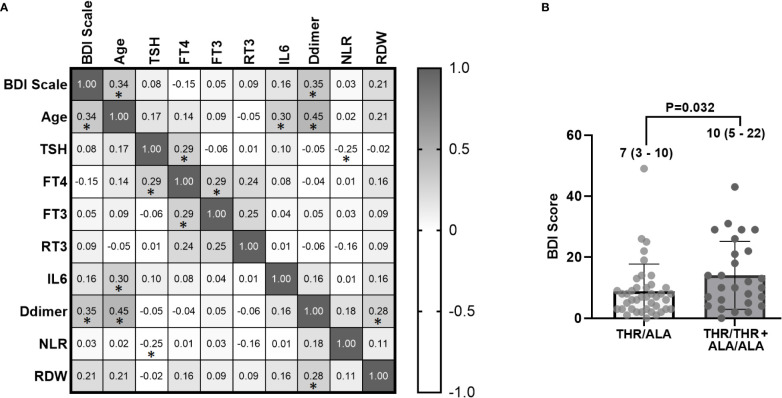
**(A)** Spearman correlation between laboratory variables of 68 hospitalized patients with COVID-19 collected within the first 48 h of admission and BDI scale scores after 30 to 45 days post-hospital discharge (* indicates *p* < 0.01). The numbers represent the correlation coefficient (*r* values). **(B)** Bar chart demonstrating higher BDI scores in homozygous patients (Thr/Thr + Ala/Ala) compared to heterozygous individuals (Thr/Ala) (*p* = 0.032). BDI, Beck Depression Inventory; TSH, thyroid-stimulating hormone; fT4, free tetraiodothyronine; fT3, free triiodothyronine; rT3: reverse triiodothyronine; IL-6, interleukin-6; NLR, neutrophil–lymphocyte ratio; RDW, red cell distribution width.

Spearman correlation analysis (**Figure 2A**) revealed a direct correlation of BDI scores with age (*r* = 0.34, *p* = 0.005) and D-dimer (*r* = 0.35, *p* = 0.003). The strongest correlation was between D-dimer versus age (*r* = 0.45, *p* < 0.0001) and D-dimer versus IL-6 (*r* = 0.30, *p* < 0.01). The BDI scores, THs, and IL-6 showed no significant difference (**Figure 2A**).

Regarding the BDI score, heterozygous patients (Thr92Ala) had lower scores than homozygous patients (*p* = 0.032) (**Table 1** and **Figure 2B**).

**Figure 3:**
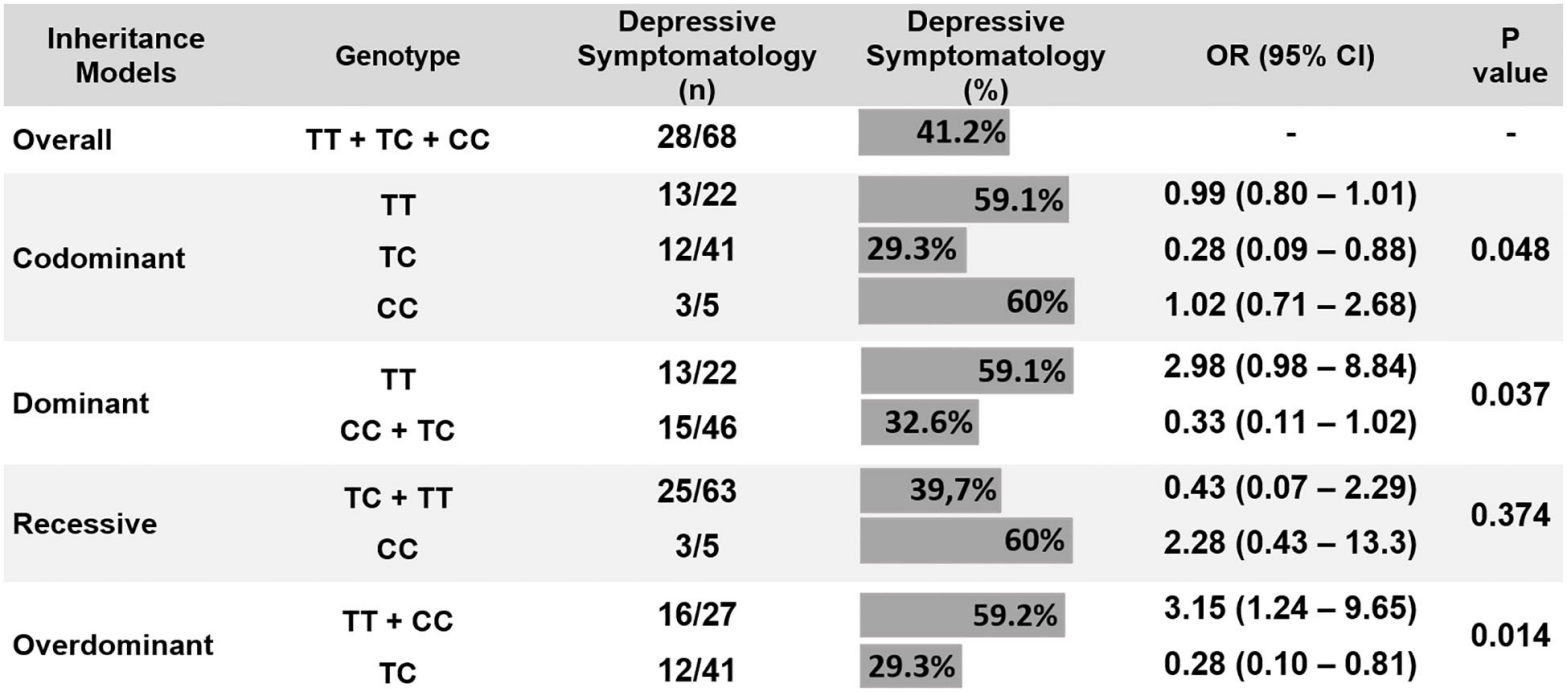
Correlation between DIO2 Thr92Ala polymorphism and depressive symptomatology (chi-square test). CI, confidence interval; OR, odds ratio.

The overall prevalence of depressive symptoms post-discharge was 41.2% (28 patients). Depressive symptoms were less common in Ala/Thr patients (29.3%) compared to Thr/Thr patients (59.1%) or Ala/Ala patients (60%) (*p* = 0.048) (**Figure 3**). Logistic regression analysis, adjusted for 15 comorbidities and other covariates, indicated that the Thr/Ala allele was associated with a reduced risk of depressive symptoms compared to the combined Thr/Thr + Ala/Ala genotype (overdominant model) (**Table 2**).

Thyroid function tests, markers of inflammation, tissue damage, and hemochromocytometric parameters are shown in **Table 2**. There was no significant difference between laboratory parameters of the patient genotypes (**Table 3**). The only laboratory variable significantly different in patients with depressive symptoms was D-dimer levels, which were higher in these patients (*p* < 0.01).

**Table 2:** Blood biochemistry in patients with COVID-19 and their association with Thr92Ala-DIO2 polymorphism and depressive symptomatology (*n* = 68).

Variables (normal range)	Total	Thr/Ala	Thr/Thr + Ala/Ala	*p*-value	Non-depression	Depression	*p*-value
	(*n* = 68)	(*n* = 41)	(*n* = 27)		(*n* = 40)	(*n* = 28)	
**TSH (0.4–5.8 µIU/mL)**	1.0 (0.65–2.25)	1.0 (0.58–2.05)	1.0 (0.82–3)	0.239	1.0 (0.65–1.89)	1.0 (0.64–3.1)	0.401
**fT3 (2.0–4.2 pg/mL)**	3.0 (2.6–3.6)	3.1 (2.6–3.7)	2.91 (2.3–3.2)	0.184	3.0 (2.59–3.7)	3.0 (2.65–3.47)	0.894
**fT4 (0.89–1.72 ng/dL)**	1.24 (0.99–1.62)	1.21 (0.98–1.6)	1.28 (1.06–1.58)	0.642	1.25 (0.99–1.62)	1.24 (0.97–1.62)	0.698
**rT3 (0.1–0.35 ng/mL)**	0.54 (0.31–0.65)	0.58 (0.29–0.68)	0.51 (0.35–0.62)	0.701	0.51 (0.29–0.61)	0.58 (0.35–0.73)	0.144
**IL-6 (<3.4 pg/mL)**	38.2 (19.4–75.1)	48.3 (18.6–69)	32.8 (19.1–83)	0.943	36.7 (17.8–67)	52.3 (27.2–77.7)	0.215
**CRP (<5.0 mg/dL)**	82.2 (48–177)	94.4 (51–179)	79.4 (45–146)	0.499	88.9 (48.7–170)	63.3 (45–177)	0.760
**Neutrophil (1.9–6.7 10³ cells/µL)**	6.74 (5.16–10.5)	7.09 (5.39–12.1)	6.64 (4.95–9.01)	0.445	7.46 (5.35–12.2)	6.68 (4.99–8.16)	0.447
**D-dimer (<500 ng/mL)**	699 (468–1,422)	696 (470–1,446)	702 (462–1,441)	0.970	**643 (384–835)**	**1,106 (615–2,824)**	**0.0016**
**LDH (207–414 U/L)**	812 (617–1,033)	807 (617–984)	815 (611–1,115)	0.644	758 (616–896)	911 (617–1,122)	0.258
**Albumin (3.5–5.5 g/dL)**	3.3 (3–3.6)	3.3 (2.9–3.6)	3.3 (3–3.6)	0.327	3.4 (3.0–3.7)	3.2 (2.9–3.6)	0.106
**HbA1c (4%–5.6%)**	7.2 (6.6–8.8)	7.4 (6.6–8.8)	7.2 (6.5–9.3)	0.986	7.1 (6.4–8.6)	7.4 (7.0–9.6)	0.111
**MCV (82–100 fL)**	89.8 (86–92)	89.4 (86–92)	90 (86–92)	0.426	89.7 (86–92)	90 (87–92)	0.889
**N/L ratio** (1–3)	9.16 (6.4–14)	9.11 (6.2–14.1)	9.4 (6.5–12.3)	0.815	9.3 (6.1–14)	9 (6.6–13.8)	0.894
**Creatinine (mg/dL)**	1.06 (0.87–1.35)	1.13 (0.89–1.37)	1.00 (0.82–1.27)	0.261	1.06 (0.89–1.3)	0.98 (0.78–1.4)	0.342

Mann–Whitney test was performed for continuous variables. CI, confidence interval; CRP, C-reactive protein; fT3, free triiodothyronine; HbA1c, hemoglobin A1c; IL-6, interleukin 6; IQR, interquartile range; LDH, lactate dehydrogenase; MCV, mean corpuscular volume; N/L ratio, neutrophil–lymphocyte ratio; OR, odds ratio; rT3, reverse triiodothyronine; TSH, thyroid-stimulating hormone.The bold values correspond to the results assessed with significant p-values (P < 0.01).

**Table 3:** Multivariable regression analyses between D2 Thr92Ala polymorphism (Thr/Thr, Thr/Ala, Ala/Ala, and overdominant model) and depressive symptomatology.

				Ala/Thr vs. Ala/Ala + Thr/Thr (Overdominant model)		
				Depression		
				OR	95% CI	*p*
Model 5 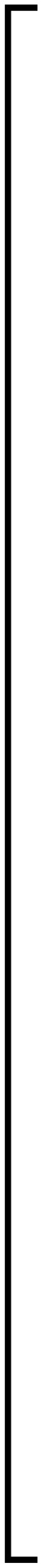	Model 4 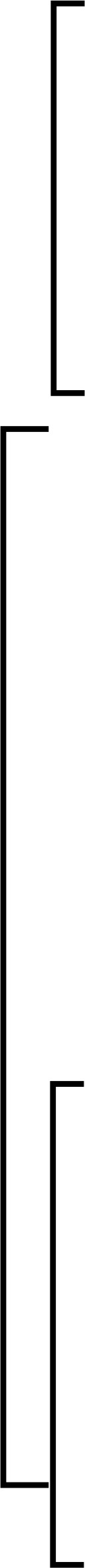	Model 1 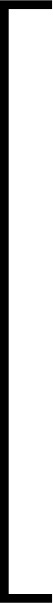	Depression	**0.28**	**0.10–0.77**	**0.015**
			Age > 60 years	**0.27**	**0.08–0.79**	**0.019**
			Gender (male)	**0.29**	**0.09–0.87**	**0.030**
			Diabetes	**029**	**0.10–0.80**	**0.018**
			SAH	**0.28**	**0.10 - 0.79**	**0.018**
			Obesity	**0.29**	**0.10– 0.79**	**0.017**
			**Model 1**	**0.28**	**0.08–0.91**	**0.039**
		Model 2 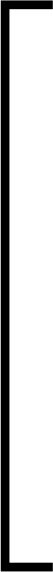	TSH	**0.30**	**0.10–0.82**	**0.022**
			Free T3	**0.27**	**0.09–0.75**	**0.014**
			Free T4	**0.27**	**0.09–0.75**	**0.013**
			Reverse T3	**0.25**	**0.08–0.70**	**0.010**
			**Model 2**	**0.22**	**0.07–0.66**	**0.009**
		Model 3 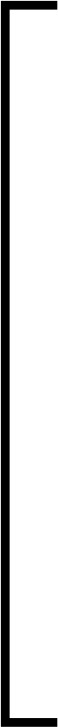	IL-6	**0.27**	**0.09–0.75**	**0.013**
			CRP	**0.29**	**0.09–0.88**	**0.031**
			D-dimer	**0.26**	**0.08–0.74**	**0.014**
			LDH	**0.24**	**0.08–0.69**	**0.009**
			Albumin	**0.30**	**0.11–0.84**	**0.024**
			Hemoglobin	**0.22**	**0.06–0.66**	**0.009**
		**Model 3**	**0.19**	**0.05–0.71**	**0.018**

Multivariable regression analyses: Model 1—adjusted for age > 60 years, diabetes, SAH, Systemic Arterial Hypertension, and obesity; Model 2—adjusted for TSH, fT3, fT4, and rT3; Model 3 - adjusted for IL6, CRP, Ddimer, Lactate dehydrogenase (LDH), albumin, and hemoglobin. Model 4—adjusted for Models 1 and 3; Model 5—adjusted for all of the above variables.The bold values correspond to the results assessed with significant p-values (P < 0.01).

## 4 Discussion

To our knowledge, this study is the first of its kind to prospectively analyze the relationship between the Thr92Ala-DIO2 polymorphism and post-COVID-19 depression in hospitalized patients. Our findings indicate that the Thr/Ala genotype correlates with a significantly reduced risk of post-discharge depression, with risk reduction ranging between 70% and 81% as per univariate and multivariate logistic regression analyses adjusted for various covariates.

DIO2, essential for physiological function in the CNS, brown adipose tissue (25), and muscle (26), plays a pivotal role in local triiodothyronine (T3) production, influencing neurological development and function. Active T3 is produced within the brain by DIO2, predominantly by astrocytes, affecting genes associated with neuronal development, myelination, and synaptic transmission (27, 28). Notably, studies on mice that lack DIO2 revealed reduced brain T3 content with mild neurological effects, such as altered emotional behaviors and memory processing. Upregulation of DIO2 has been observed in various neurological disorders, influencing gene expression associated with inflammation and cell death.

The Thr92Ala D2 polymorphism has been associated with decreased TH activity in various end-organ targets. Research conducted *in vitro* and *ex vivo* suggests that the Ala allele is linked to enzyme dysfunction, impacting neurodegenerative mechanisms and oxidative stress within the central nervous system (29). Additionally, this polymorphism has been correlated with various neuropsychiatric conditions, including autism (Marcondes et al., 2021), schizophrenia (30), depression (19, 20), and cognitive impairment (31).

A Lithuanian study involving 168 participants investigated the link between 10 single-nucleotide polymorphisms (SNPs) in DIO1, DIO2, DIO3, and transmembrane TH transporters, specifically the organic anion transporter polypeptide 1C1 (OATP1C1), in relation to post-stroke depressive symptoms and anxiety. Among these SNPs, only the wild-type OATP1C1-rs974453 genotype (GG) showed a significant association with an increased likelihood for depression symptoms (OR = 2.73; 95% CI: 1.04–7.12; *p* = 0.041). In contrast, the Thr92Ala polymorphism did not demonstrate a statistically significant difference, even though it was more prevalent in the Thr/Thr genotype in patients with depression (20). Conversely, a study in Poland indicated that the Ala–Ala genotype of the Thr92Ala polymorphism was more common in healthy individuals compared to those with recurrent depression (7.2% vs. 0.6%, *p* = 0.03, respectively), suggesting its potential as a marker for reduced risk of recurrent depressive disorder (32).

This protection may be explained by the association of Thr92Ala-DIO2 gene expression with endoplasmic reticulum (ER) stress, inflammation, oxidative stress, apoptosis, and mitochondrial dysfunction (33). Disruption of ER homeostasis can lead to the accumulation of misfolded or unfolded proteins in the ER lumen, a condition referred to as ER stress. ER stress is associated with obesity, insulin resistance, type 2 diabetes (34), endothelial dysfunction (35), and low-grade chronic inflammation (36). These conditions have been associated with higher risk and worse prognosis of COVID-19 (37) and depression (38, 39).

Clinical studies have found associations between decreased levels of brain-derived neurotrophic factor (BDNF) and increased inflammatory markers, which are linked to the onset of depressive symptoms and various psychiatric disorders (40). In individuals with depression, one study revealed a correlation between elevated TSH levels, decreased serum BDNF levels, and a lower rise in BDNF during antidepressant treatment (41). A more recent study examining 50 patients undergoing their first episode of psychosis showed that high TSH levels were associated with low peripheral BDNF and reduced hippocampal volume, suggesting a potential neuroprotective effect of THs on the hippocampus (42).

Animal studies also contribute to our understanding by showing that BDNF has a protective effect against ER stress-induced cell death in brain neurons. This mechanism, which depends on PI3-K activation and inhibits caspase-12, highlights the importance of BDNF in maintaining neuronal integrity under stress (43). The connection between the Thr92Ala-DIO2 gene, ER stress, and BDNF regulation provides a comprehensive perspective on the biological mechanisms that protect against pathological conditions and depressive symptoms.

Research indicates a substantial genetic factor in depression, estimated to contribute approximately 30%–40% to its heritability (44, 45). A recent study found a significant link between the SIRT1 rs12415800 polymorphism, a gene associated with longevity, cellular defense against oxidative stress, and depressive symptoms in university students (46). This association was evident in both codominant (*p* = 0.0437) and overdominant (*p* = 0.0147) genetic models, demonstrating the heterozygous advantage (similar to our study) of this polymorphism against depressive symptoms (47).

Microglial cells, specific types of macrophages in the central nervous system, play a crucial role in neuroinflammation and are increasingly linked to the development of depression. Recent findings suggest a potential link between depression onset and viral infections like SARS-CoV-2, BoDV-1, ZIKV, HIV, and HHV6, which impact various glial cells, including astrocytes, oligodendrocytes, and microglia (48). Transcriptomic analysis [Gene Set Enrichment Analysis (GSEA)] of mice with the Thr92Ala polymorphism revealed increased gene expression related to neuroplasticity, cognition, apoptosis, and neuroinflammation. These results strongly suggest an association between Thr92Ala and neuroinflammation, involving astrocytes as the primary cell type expressing DIO2 in the central nervous system (49).

There are some limitations in this study. Our sample size was relatively small, and the observation time was short; we did not collect healthy people as controls, which may have some influence on the study results; we used mostly self-assessment scales, which may introduce recall bias; and we were unable to measure serum BDNF levels in our patients.

In this prospective study, we present evidence suggesting that possessing heterozygosity of Thr92Ala-DIO2 may have a protective role in preventing the occurrence of depressive symptoms after being discharged from the hospital. Additional research is needed to confirm these findings.

## Data availability statement

The data presented in the study have been deposited in the figshare repository with the following accession number: https://doi.org/10.6084/m9.figshare.25118159.v1.

## Ethics statement

The studies involving humans were approved by Hospital Universitario Lauro Wanderley’s Ethics Committee for Human Research. The studies were conducted in accordance with the local legislation and institutional requirements. The participants provided their written informed consent to participate in this study.

## Author contributions

DB: Data curation, Investigation, Methodology, Writing – original draft. FEB: Formal analysis, Investigation, Methodology, Software, Writing – original draft. GC: Data curation, Methodology, Writing – review & editing. FLB: Data curation, Methodology, Writing – review & editing. AB: Data curation, Writing – review & editing. HS: Data curation, Methodology, Writing – review & editing. HT: Data curation, Methodology, Writing – review & editing. JR: Data curation, Methodology, Writing – review & editing. CF: Data curation, Writing – review & editing. RC: Conceptualization, Validation, Writing – review & editing. LP: Investigation, Writing – review & editing, Methodology. GV: Conceptualization, Writing – review & editing. HR: Conceptualization, Investigation, Project administration, Supervision, Writing – original draft, Writing – review & editing.

## Supplementary material

The Supplementary Material for this article can be found onlineat: https://www.frontiersin.org/articles/10.3389/fendo.2024.1366500/full#supplementary-material
