# Supplementary material for: The Thr92Ala polymorphism in the type 2 deiodinase gene is linked to depression in patients with COVID-19 after hospital discharge
Source: Front Endocrinol (Lausanne). 2024 Jun 7;15:1366500. doi: 10.3389/fendo.2024.1366500 (PMC11190161; doi:10.3389/fendo.2024.1366500)
Supplement: Supplementary file 1 [file Table_1.docx]

**Supplementary table 1**. Baseline Patient Characteristics

| Patients | rs225014 | Beck's Depression Inventory | Depression | **Sex** | **Age** | **length of hospital stay** | **Gravity** | **TSH** | **Free T4** | **Free T3** | **Reverse T3** |
| --- | --- | --- | --- | --- | --- | --- | --- | --- | --- | --- | --- |
| 1 | Heterozygous C/T | 2 | No | M | 64 | 10 | Critical | 1,41 | 1,36 | 2,55 | 0,22 |
| 2 | Heterozygous C/T | 9 | No | M | 45 | 8 | Critical | 2,35 | 0,98 | 3,31 | 0,28 |
| 3 | Heterozygous C/T | 8 | No | M | 60 | 4 | Non-Critical | 3,62 | 1,47 | 3,67 | 0,51 |
| 4 | Heterozygous C/T | 3 | No | M | 42 | 6 | Non-Critical | 2,30 | 0,67 | 2,03 | 0,55 |
| 5 | Heterozygous C/T | 6 | No | F | 43 | 13 | Non-Critical | 2,64 | 1,21 | 1,51 | 0,42 |
| 6 | Homozygous T/T | 3 | No | M | 35 | 2 | Non-Critical | 0,99 | 1,22 | 2,3 | 0,51 |
| 7 | Heterozygous C/T | 8 | No | F | 54 | 3 | Non-Critical | 2,10 | 1,69 | 4,55 | 0,85 |
| 8 | Heterozygous C/T | 5 | No | F | 80 | 3 | Non-Critical | 0,36 | 1,15 | 2,96 | 0,5 |
| 9 | Heterozygous C/T | 3 | No | M | 75 | 7 | Non-Critical | 1,00 | 2,26 | 3,2 | 0,47 |
| 10 | Homozygous T/T | 8 | No | M | 82 | 5 | Non-Critical | 0,81 | 2,01 | 3,71 | 0,57 |
| 11 | Heterozygous C/T | 1 | No | M | 78 | 2 | Non-Critical | 1,03 | 1,34 | 2,8 | 0,6 |
| 12 | Heterozygous C/T | 6 | No | M | 30 | 7 | Non-Critical | 1,00 | 2,49 | 3,8 | 1 |
| 13 | Heterozygous C/T | 2 | No | M | 40 | 7 | Non-Critical | 1,00 | 1,94 | 3,05 | 0,59 |
| 14 | Heterozygous C/T | 3 | No | M | 47 | 8 | Non-Critical | 0,19 | 1,38 | 2,61 | 0,5 |
| 15 | Heterozygous C/T | 5 | No | M | 53 | 9 | Non-Critical | 2,98 | 1,49 | 2,59 | 0,29 |
| 16 | Homozygous C/C | 0 | No | M | 32 | 5 | Non-Critical | 0,44 | 0,63 | 2,75 | 0,57 |
| 17 | Heterozygous C/T | 9 | No | M | 78 | 12 | Non-Critical | 0,63 | 0,79 | 3,18 | 0,68 |
| 18 | Heterozygous C/T | 0 | No | M | 42 | 4 | Non-Critical | 0,90 | 1,21 | 2,81 | 0,34 |
| 19 | Heterozygous C/T | 2 | No | M | 64 | 6 | Non-Critical | 0,63 | 2,24 | 4,1 | 0,66 |
| 20 | Heterozygous C/T | 3 | No | M | 42 | 5 | Non-Critical | 1,44 | 0,94 | 2,88 | 0,22 |
| 21 | Heterozygous C/T | 7 | No | M | 51 | 5 | Non-Critical | 1,33 | 1,47 | 3,79 | 0,59 |
| 22 | Heterozygous C/T | 7 | No | M | 46 | 4 | Non-Critical | 0,71 | 1,2 | 2,79 | 0,29 |
| 23 | Heterozygous C/T | 3 | No | F | 53 | 8 | Non-Critical | 1,32 | 0,98 | 2,51 | 0,22 |
| 24 | Heterozygous C/T | 6 | No | M | 54 | 7 | Non-Critical | 1,62 | 1,31 | 3,4 | 0,69 |
| 25 | Heterozygous C/T | 8 | No | M | 79 | 6 | Non-Critical | 0,81 | 0,94 | 2,68 | 0,11 |
| 26 | Heterozygous C/T | 2 | No | M | 48 | 5 | Non-Critical | 1,79 | 1,01 | 4,12 | 0,21 |
| 27 | Heterozygous C/T | 8 | No | M | 36 | 5 | Non-Critical | 0,74 | 0,9 | 2,55 | 0,24 |
| 28 | Homozygous T/T | 4 | No | M | 43 | 10 | Non-Critical | 0,18 | 1,08 | 2,93 | 0,51 |
| 29 | Homozygous T/T | 2 | No | M | 47 | 5 | Non-Critical | 3,00 | 2,29 | 4 | 0,83 |
| 30 | Homozygous C/C | 2 | No | M | 41 | 4 | Non-Critical | 3,11 | 1,28 | 1,57 | 0,64 |
| 31 | Homozygous T/T | 5 | No | M | 55 | 3 | Non-Critical | 0,82 | 1,08 | 2,69 | 0,31 |
| 32 | Heterozygous C/T | 1 | No | M | 66 | 5 | Non-Critical | 3,42 | 1,42 | 3,78 | 0,65 |
| 33 | Homozygous T/T | 6 | No | F | 54 | 5 | Non-Critical | 0,88 | 1,5 | 3,18 | 0,45 |
| 34 | Homozygous T/T | 4 | No | F | 59 | 19 | Non-Critical | 1,00 | 1,99 | 3,05 | 0,14 |
| 35 | Heterozygous C/T | 4 | No | M | 49 | 7 | Non-Critical | 0,30 | 1,67 | 3,4 | 0,59 |
| 36 | Homozygous T/T | 7 | No | M | 55 | 12 | Non-Critical | 1,93 | 1,81 | 4,17 | 0,9 |
| 37 | Heterozygous C/T | 8 | No | M | 41 | 4 | Non-Critical | 0,54 | 0,58 | 5,19 | 0,41 |
| 38 | Homozygous T/T | 7 | No | M | 53 | 10 | Non-Critical | 1,07 | 1,02 | 1,17 | 0,58 |
| 39 | Heterozygous C/T | 7 | No | M | 42 | 21 | Non-Critical | 0,12 | 1,2 | 3,14 | 0,19 |
| 40 | Heterozygous C/T | 3 | No | M | 53 | 6 | Non-Critical | 0,30 | 0,99 | 2,5 | 0,61 |
| 41 | Heterozygous C/T | 14 | Yes | M | 50 | 22 | Critical | 0,74 | 1,1 | 2,94 | 0,84 |
| 42 | Homozygous C/C | 31 | Yes | M | 68 | 18 | Critical | 1,11 | 1,06 | 2,14 | 0,35 |
| 43 | Homozygous T/T | 14 | Yes | F | 72 | 34 | Critical | 6,30 | 1,44 | 2,91 | 0,36 |
| 44 | Homozygous T/T | 18 | Yes | F | 75 | 5 | Critical | 0,54 | 1,48 | 2,8 | 0,4 |
| 45 | Heterozygous C/T | 22 | Yes | F | 79 | 8 | Critical | 2,00 | 1,78 | 3,1 | 0,29 |
| 46 | Heterozygous C/T | 14 | Yes | M | 36 | 4 | Non-Critical | 0,81 | 1,21 | 3,3 | 6,1 |
| 47 | Homozygous T/T | 29 | Yes | M | 66 | 5 | Non-Critical | 0,45 | 1,42 | 3,8 | 0,73 |
| 48 | Heterozygous C/T | 10 | Yes | M | 72 | 7 | Non-Critical | 0,61 | 1,27 | 2,63 | 0,58 |
| 49 | Homozygous T/T | 10 | Yes | F | 72 | 3 | Non-Critical | 3,70 | 1,7 | 3,91 | 0,13 |
| 50 | Homozygous T/T | 12 | Yes | F | 67 | 3 | Non-Critical | 3,00 | 1,38 | 2,36 | 0,84 |
| 51 | Homozygous T/T | 13 | Yes | M | 50 | 10 | Non-Critical | 1,00 | 1,5 | 3,2 | 0,62 |
| 52 | Homozygous C/C | 26 | Yes | M | 67 | 5 | Non-Critical | 3,66 | 0,8 | 2,19 | 0,49 |
| 53 | Heterozygous C/T | 10 | Yes | M | 65 | 3 | Non-Critical | 0,38 | 0,61 | 4,31 | 0,69 |
| 54 | Homozygous T/T | 21 | Yes | M | 64 | 6 | Non-Critical | 0,81 | 0,94 | 2,68 | 0,11 |
| 55 | Homozygous T/T | 10 | Yes | F | 68 | 1 | Non-Critical | 1,55 | 1,12 | 2,64 | 0,61 |
| 56 | Heterozygous C/T | 19 | Yes | M | 61 | 7 | Non-Critical | 3,10 | 1,7 | 1,95 | 0,75 |
| 57 | Homozygous T/T | 29 | Yes | M | 67 | 3 | Non-Critical | 1,18 | 1,07 | 2,84 | 0,58 |
| 58 | Heterozygous C/T | 11 | Yes | M | 42 | 5 | Non-Critical | 0,20 | 1,68 | 3,09 | 0,77 |
| 59 | Heterozygous C/T | 10 | Yes | F | 45 | 5 | Non-Critical | 0,99 | 1,06 | 2,91 | 0,88 |
| 60 | Heterozygous C/T | 25 | Yes | F | 77 | 3 | Non-Critical | 5,66 | 1,71 | 3,22 | 0,59 |
| 61 | Heterozygous C/T | 13 | Yes | F | 74 | 42 | Non-Critical | 4,00 | 1,64 | 4 | 0,63 |
| 62 | Homozygous T/T | 29 | Yes | F | 77 | 6 | Non-Critical | 0,88 | 0,87 | 3,99 | 0,35 |
| 63 | Homozygous T/T | 22 | Yes | F | 59 | 12 | Non-Critical | 2,56 | 1,08 | 2,94 | 0,71 |
| 64 | Homozygous T/T | 14 | Yes | F | 57 | 7 | Non-Critical | 0,99 | 0,92 | 2,3 | 0,5 |
| 65 | Heterozygous C/T | 49 | Yes | F | 52 | 6 | Non-Critical | 0,56 | 0,93 | 3,53 | 0,23 |
| 66 | Homozygous T/T | 43 | Yes | M | 45 | 6 | Non-Critical | 3,64 | 1,58 | 3,11 | 0,31 |
| 67 | Heterozygous C/T | 26 | Yes | F | 55 | 12 | Non-Critical | 0,38 | 0,31 | 4,18 | 0,74 |
| 68 | Homozygous C/C | 10 | Yes | M | 59 | 7 | Non-Critical | 1,00 | 2,06 | 3,22 | 0,53 |

**Supplementary table 2**. Primers for allele-specific transcript quantification by NGS. (Bsg-1- New England BioLabs, Inc.)

| rs225014 Sense primer (21 nt) | 5’- CTCAGGGCTGGCAAAGTCAAG-3’ |
| --- | --- |
| rs225014 Antisense primer (22nt) | 5’- CCACACTCTATTAGAGCAATTG-3’ |
